# Association of Vitamin D Intake, *VDR* Levels, and Relevant Gene Polymorphisms in Early-Stage Thai Colorectal Cancer Patients Following Vitamin D_3_ Supplementation

**DOI:** 10.3390/medsci14050518

**Published:** 2026-08-26

**Authors:** Natthaporn Kuendee, Aunchana Rodrungnok, Kamphon Intharanut, Alisa Naladta, Oytip Nathalang, Sakda Daduang, Sophida Sukprasert

**Affiliations:** 1 Faculty of Veterinary Medicine, Rajamangala University of Technology Tawan-ok, Chonburi 20110, Thailand; natthaporn_ku@rmutto.ac.th; 2 Chulabhorn International College of Medicine, Thammasat University (Rangsit Campus), Pathum Thani 12120, Thailand; ard@tu.ac.th; 3 Thammasat University Research Unit in Integrative and Translational Medicine, Thammasat University (Rangsit Campus), Pathum Thani 12120, Thailand; alisa_n@kkumail.com; 4 Graduate Program in Biomedical Sciences, Faculty of Allied Health Sciences, Thammasat University (Rangsit Campus), Pathum Thani 12120, Thailand; kamphon.int@gmail.com (K.I.); oytipntl@hotmail.com (O.N.); 5 Division of Pharmacognosy and Toxicology, Faculty of Pharmaceutical Sciences, Khon Kaen University, Khon Kaen 40002, Thailand; sakdad@kku.ac.th; 6 Protein and Proteomics Research Center for Commercial and Industrial Purposes (ProCCI), Khon Kaen University, Khon Kaen 40002, Thailand

**Keywords:** colorectal cancer, CYP27B1, CYP24A1, vitamin D receptor, polymorphisms

## Abstract

Background: Recent studies have reported associations between vitamin D receptor (*VDR*) gene polymorphisms and colorectal cancer (CRC). However, the findings thus far are inconsistent. This study aims to clarify the association of vitamin D intake, *VDR* levels, and relevant polymorphisms in early stages of CRC in Thai patients following vitamin D_3_ supplementation. Methods: Total RNA and genomic DNA were isolated from the buffy coats of CRC patients (*n* = 27). The levels of *VDR*, *RXR*, *CYP27B1*, and *CYP24A1* were measured using quantitative real-time PCR. Associations of nine single-nucleotide polymorphisms (SNPs) of *VDR*, *CYP27B1*, *CYP24A1* and serum vitamin D levels were investigated. Results: The expression levels of *VDR*, *RXR* and *CYP27B1* were significantly elevated in the vitamin D_3_-treated group. However, no significant associations were observed between vitamin D intake and the specific *VDR* SNPs, including FokI, BsmI, ApaI, TaqI, and Tru9I. Furthermore, none of the SNPs or haplotypes within the *VDR*, *CYP27B1* (rs10877012), or *CYP24A1* (rs6013897, rs158552, rs17217119) were linked to colorectal cancer susceptibility. Conclusions: This finding demonstrated that vitamin D intake enhances the expression of *VDR* and related genes in early-stage CRC patients; these effects are independent of *VDR*, *CYP27B1*, and *CYP24A1* genetic polymorphisms. This suggests that gene expression changes may be driven more by vitamin D status than by underlying genetic variation.

## 1. Introduction

Colorectal cancer (CRC) is the second leading cause of cancer-related death and the third most diagnosed cancer worldwide [1]. In Thailand, it is the fourth most frequently diagnosed cancer, with an incidence of 11.4% in males and 10.7% in females [2]. According to the Thai cancer registry data expected for the year 2025, CRC is continuing to increase nationwide, with an average annual percent change (AAPC) of 3.3% and 4.1% for females and males, respectively [3]. Vitamin D_3_ (VD_3_) is a crucial form of vitamin D, a fat-soluble secosteroid produced in the skin from 7-dehydrocholesterol when exposed to ultraviolet B radiation. It can also be sourced from dietary intake and supplements. VD_3_ is subsequently hydroxylated in the liver to form 25-hydroxyvitamin D_3_ [25(OH)D_3_] and further hydroxylated in the kidney to produce the biologically active hormone 1,25-dihydroxy-vitamin D_3_ [1,25(OH)_2_D_3_], which exerts its biological effects primarily through the vitamin D receptor (VDR) [4]. Non-classical inhibitory mechanisms for the growth of VD_3_ in cancer have been reported. The active 1,25(OH)_2_D_3_ (calcitriol, CAL) of VD_3_ can inhibit cell proliferation, invasion, metastasis, and angiogenesis, inducing apoptosis and stabilizing intact chromosome structure through modification of innate and adaptive immune activity [4,5,6]. Moreover, it plays a role in the maintenance of immune homeostasis and inhibits cancer growth in CRC [7,8]. Supplementation with VD_3_ effectively suppresses intestinal tumorigenesis in animal models [9,10,11]. VD_3_ has been estimated to lower the incidence of CRC by 50%, which is consistent with the inverse correlation between dietary VD_3_ intake or sunlight exposure and human CRC [12]. Several studies have confirmed that increasing calcitriol levels lowers CRC incidence and reduces polyp recurrence and that sufficient levels of CAL are associated with improved overall survival of CRC patients [12,13]. In the cellular mechanism, VD_3_ is hydroxylated by the *CYP27B1* gene, which encodes a 1α-hydroxylase to generate an active form of CAL. CAL subsequently binds to the nuclear vitamin D receptor (VDR), which heterodimerizes with the retinoid X receptor (RXR) to interact with the vitamin D responsive element to control transcription [14]. Additionally, the enzyme 1,25-dihydroxyvitamin D_3_-24-hydroxylase, which is encoded by the *CYP24A1* gene, is responsible for breaking down CAL by converting it to less active or inactive metabolites [15].

Recently, the associations between vitamin D and CRC have received considerable attention, and studies on genetic factors influencing vitamin D metabolism have been actively conducted [16,17]. Numerous candidate gene studies and genome-wide association studies identified a series of genetic mutations and polymorphisms affecting the gene encoding the molecules involved in the production and activation of vitamin D [18]. The most investigated genes are *VDR*, *CYP27B1*, and *CYP24A1*. The *VDR* gene is located on chromosome 12 (12q13.11) with six promoter regions. Four types of *VDR* polymorphisms, consisting of TaqI, BsmI, ApaI, and FokI, which are recognized by their restriction endonuclease enzymes [19], are associated with serum levels of 25-hydroxy vitamin D (calcifediol, CALF, (25(OH)D). FokI rs10735810, also known as rs2228570 C>T, is located in exon 2; the T nucleotide is referred to as allele “f” while the C nucleotide is defined as allele “F”. The presence of site “F” results in a three-amino-acid-shortened protein, which is characterized by increased transcriptional activity. In addition, the “f” allele is associated with lower serum CALF levels. TaqI (rs731236 T>C) is located in exon 9; the T nucleotide is defined as allele “T”, while the C nucleotide corresponds to allele “t”. This polymorphism occurs in a CpG island, resulting in an influence on the methylation status. BsmI (rs1544410 A>G) is located in intron 8; the “A” nucleotide corresponds to allele “B,” and the “G” nucleotide corresponds to allele “b”. It influences the variation in the UVB-induced CALF increase, associated with the interaction with *RXRA* and *CYP24A1* [18,20]. ApaI (rs7975232 C>A) is located in intron 8; the functional impact of this polymorphism is still not clearly explained [18]. Furthermore, the *CYP27B1* gene (25(OH)D-1α-hydroxylase rs10877012) is located on chromosome 12 and encodes the important 1α-hHydroxylase, which converts CALF to the active form CAL [21]. This SNP is situated in the promoter region of the *CYP27B1* gene and is shown to be associated with reduced serum levels of CALF [18,22]. In addition, the *CYP24A1* gene (rs17219315) is located on chromosome 20, represented as a mitochondrial enzyme expressed in several target cells containing VDRs, which catalyzes CALF and CAL catabolism and was associated with serum CALF concentration.

Several studies have demonstrated associations between single-nucleotide polymorphisms (SNPs) and vitamin D metabolism, including serum CALF levels and related metabolic traits [16,17,18,19,21,22,23]. Evidence also suggests that vitamin D supplementation can improve metabolic factors, enhance serum vitamin D status, and modulate immune responses in CRC patients [24]. In this regard, the effects of high-dose vitamin D intake and the cellular actions of VD_3_ in CRC may in part be influenced by variations in vitamin D levels associated with specific SNPs. Therefore, the present study aimed to investigate the relationships among vitamin D intake, *VDR* expression, and relevant genetic polymorphisms of *VDR*, including *CYP27B1* and *CYP24A1*, in the early stages of CRC in Thai patients following VD_3_ supplementation.

## 2. Materials and Methods

### 2.1. Sample

The buffy coat samples (*n* = 27) were residual specimens obtained from a previous study [24] consisting of 11 controls and 16 cases, who received 8000 IU of VD_3_ daily for 3 months. Briefly, 6 mL of fresh EDTA whole blood was collected after VD_3_ supplementation at each time point. The samples were then spun at 1200× *g* for 10 min at room temperature. Residual plasma was aspirated, and the buffy coats were situated directly above the red blood cells using a pipette. The collected samples were placed into clean sterile tubes and stored at −80 °C until use for downstream processes.

### 2.2. Total RNA Isolation and Quantitative RT-PCR (RT-qPCR)

Total RNA was isolated from buffy coats using TRI reagent (Molecular Research Center, Inc., Cincinnati, OH, USA). The solutions were then transferred to a QIAamp Mini Spin Column (QIAamp RNA Blood Mini Kit; QIAGEN, Hilden, Germany) according to the manufacturer’s protocol. The total RNA concentration and purity were determined by measuring the absorbance at 260 and 280 nm using a NanoDrop 2000 instrument (Thermo Scientific, Vilnius, Lithuania). RNA was transcribed into cDNA using the cDNA Synthesis Kit (Thermo Scientific, Vilnius, Lithuania). The obtained cDNA was stored at −20 °C for RT-qPCR. Primers were designed and synthesized to investigate the expression levels of *VDR*, *RXR*, *CYP27B1*, and *CYP24A1*, as shown in Table 1. RT-qPCR conditions were performed as follows: after pre-denaturation at 95 °C for 10 min, PCR was performed for 50 cycles, each of which consisted of 20 s at 94 °C, 20 s at 55 °C, and 30 s at 72 °C. Gene expression was analyzed using the 2^−∆∆Ct^ method according to a previous study [25]. *β*-Actin was used as a housekeeping gene for normalization of target gene expression. Every sample was analyzed in triplicate.

### 2.3. DNA Extraction and SNPs Genotyping by DNA Sequencing

Genomic DNA from the buffy coat samples (*n* = 27) was extracted using QIAamp DNA Mini and Blood Mini Kit (QIAGEN, Hilden, Germany) according to the manufacturer’s protocol. Sanger DNA sequencing was performed to genotype the following SNPs: *VDR* rs7975232, rs731236, rs1544410, rs757343, and rs2228570, *CYP24A1* rs6013897, rs158552, and rs17217119, and *CYP27B1* rs10877012. Specific primers were designed using Primer3Plus software (version 4.1.0) with the purpose of amplifying the targeted gene region. The primer combination sequences used in the reaction, target genes, SNPs, and product size are shown in Table 2. The unmodified DNA oligonucleotides were all purchased from U2Bio Co., Ltd. In Bangkok, Thailand with standard high-affinity purification and kept in a refrigerator at 4 °C.

The eligible genomic DNA from 27 samples was sequenced for each SNP genotyping. For the *VDR* target gene, the amplified fragment of SQ1 contained SNPs of rs7975232 (C>A) and rs731236 (A>G/T), while the amplified fragment of SQ2 contained SNPs of rs1544410 (C>A/G/T) and rs757343 (C>T), and the amplified fragment of the SQ3 primer contained SNPs of rs2228570 (A>G/C/T). In addition, the amplified fragments of SQ4 and SQ5 contained the SNPs of rs158552 (C>G/T) and rs17217119 (A>G) of the *CYP24A1* gene and rs10877012 (G>T/C) of the *CYP27B1* gene, respectively. All genes targeted during PCR amplification for DNA sequencing were the same as those in the PCR mixtures and conditions. The components used for each PCR reaction were genomic DNA (100 ng/mL), forward and reverse primers (10 pmol/mL), and a 2×PCR reaction mixture (Phusion High-Fidelity PCR Master Mix; New England BioLabs, Ipswich, MA, USA). PCR was performed on a T100 Thermal Cycler (Bio-Rad Laboratories, Inc., Hercules, CA, USA). Thermal cycling started with a 30 s incubation step at 98 °C for polymerase activation, followed by 35 cycles of 30 s at 98 °C for DNA denaturing, 40 s at 62 °C for annealing, and 30 s at 72 °C for extension, and a final extension of 5 min at 72 °C. A gel extraction kit (GeneJET Gel Extraction Kit; Thermo Scientific, Waltham, MA, USA) was then used to purify the targeted amplicons. The eluted fragments were sequenced using these PCR primers and the Sanger method (DNA Sequencing Service, U2Bio Co., Ltd., Bangkok, Thailand).

### 2.4. Statistical Analysis

The genotype and allele frequencies were determined by direct counting. The chi-square (χ^2^) test was employed to compare the observed genotype frequencies to the expected genotypes under Hardy–Weinberg equilibrium (HWE). The control and intervention groups were compared in terms of genotypes and SNP allele frequencies. The relative expression levels of *VDR*, *RXR*, *CYP27B1*, and *CYP24A1* were analyzed using One-Way ANOVA (nonparametric), followed by the Kruskal–Wallis test and Dunn’s multiple comparisons test. The data were represented as mean ± SEM. The best-fitting inheritance model is shown after genotype-wise median 2^∆Ct values (∆Ct = control Ct − gene of interest Ct) were computed based on the starting assumption of a dominant model using GraphPad InStat (GraphPad Software Inc., Version 9.5.1, San Diego, CA, USA). The distribution of expression levels by genotype was examined for differences using *p*-values derived from non-parametric analysis of variance testing (Wilcoxon signed-rank test). The differences were considered significant at *p* < 0.05.

## 3. Results

### 3.1. Expression Levels of VDR, RXR, CYP27B1, and CYP24A1

**Figure 1:**
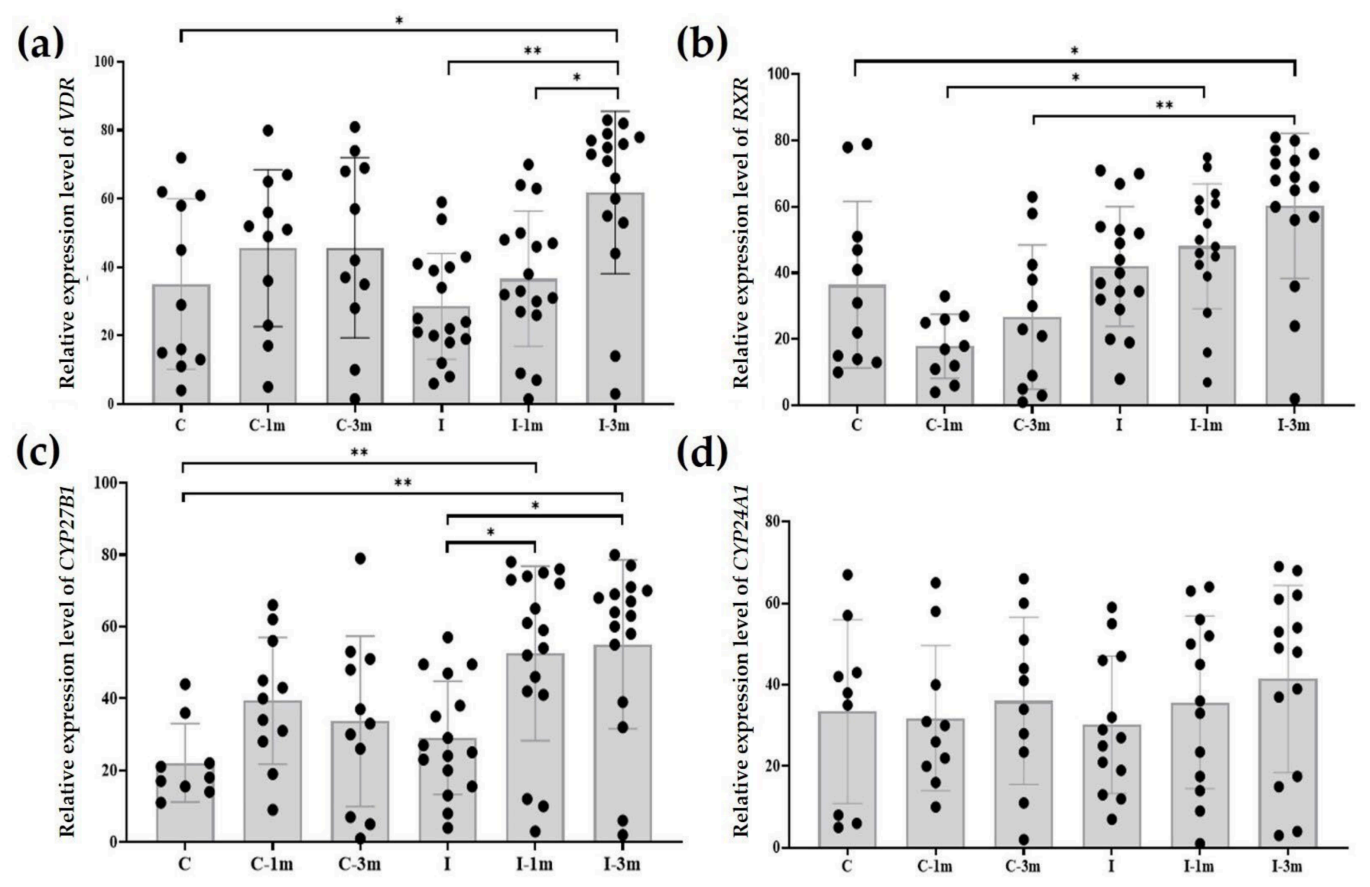
Relative expression levels of *VDR* (**a**), *RXR* (**b**), *CYP27B1* (**c**), and *CYP24A1* (**d**) in early stages of CRC patients with (I = intervention) and without (C = control) VD_3_ supplementation for 3 months. Data are represented as mean ± SEM. * and ** were significant levels at *p*-value < 0.05 and 0.01, respectively.

As shown in Figure 1, the gene expression level of *VDR* was significantly upregulated in the VD_3_-treated group after receiving VD_3_ for 3 months when compared to the control group. In the intervention group, the results revealed that *VDR* levels were highly expressed after receiving VD_3_ for 3 months (Figure 1a). Similarly, the results showed that *RXR* levels were highly upregulated in the VD_3_-treated group at 3 months when compared to the control group (Figure 1b). In accordance with *CYP27B1*, gene expression levels of *CYP27B1* were dominantly upregulated in the intervention group at 1 and 3 months when compared to within and between groups (Figure 1c). However, no significance of *CYP24A1* was observed in the VD_3_-treated group compared to the control group (Figure 1d).

### 3.2. Genetic Polymorphism of VDR, CYP27B1, and CYP24A1

The observed genotype distributions of 9 SNPs of *VDR*, *CYP27B1*, and *CYP24A1* genes were calculated to conform to the Hardy–Weinberg equilibrium (HWE) test at *p*-value > 0.05. As shown in Table 3, genetic characteristics between the intervention and control groups: 5 SNP polymorphic sites were amplified from *VDR*, while 1 and 3 SNP polymorphic sites were amplicons of the *CYP27B1* and *CYP24A1* genes, respectively.

For the *VDR*, rs731236 (TaqI) SNP, a higher frequency of mutant homozygous type TT (tt) was observed in both the intervention (87.5%) and control groups (90.9%), while heterozygous type CT (Tt) was only observed in the intervention (12.5%) and the wild-type homozygous type CC (TT) was only shown in the control group (9.09%).

Similarly, for the rs1544410 (BsmI) SNP, the mutant homozygous genotype GG (bb) was more frequent in both the intervention (75.0%) and control (90.91%) groups, while the heterozygous genotype AG (Bb) was observed only in the intervention group (25.0%), and the wild-type homozygous genotype AA (BB) was observed in the control group (9.09%). In contrast, the rs757343 (Tru9I) SNP showed no mutant homozygous TT genotypes in either group. The wild-type homozygous CC genotype was more frequent in the control group (54.54%), whereas the heterozygous CT genotype predominated in the intervention group (62.5%). As for the rs7975232 (ApaI) and rs2228570 (FokI) SNPs, a similar frequency of genotype was observed in both groups which high frequency of heterozygous AC (Aa) and CT (Ff), respectively.

When considering the *CYP27B1* rs10877012 SNP, the highest frequency was shown in the heterozygous type GT (Tt) in both groups, with 62.50% for intervention and 45.45% for control, followed by mutant homozygous gg (tt) and wild-type homozygous GG (TT), respectively. For *CYP24A1*, rs6013897 and rs17217119 showed no polymorphism in the mutant homozygous type in both groups and showed high frequency in wild-type homozygous. In contrast, the rs158522 SNP showed no polymorphism in wild-type homozygous CC and the highest frequency in mutant homozygous TT.

### 3.3. Association Between VDR, CYP27B1, CYP24A1, and SNPs

The association between the relative expression levels of all genes (*VDR*, *CYP27B1*, *CYP24A1* and SNPs was investigated (Table 4). There were no significant correlations between the SNPs and the mRNA expression levels in those samples, despite having shown that all homozygous common genotypes expressed these three genes at greater levels than the heterozygous and homozygous variant genotypes (*p* > 0.05). Therefore, the correlation between serum vitamin D levels and SNP genotypic distribution of *VDR*, *CYP27B1*, and *CYP24A1* in the VD_3_-treated group was investigated. The results revealed no association between SNP genotype and serum vitamin D level, indicating that the SNP genotype was not correlated with the increasing of serum vitamin D level at 3 months after supplementation of high-dose VD_3_ 8000 IU daily when compared with their baseline values [24].

## 4. Discussion

The pathophysiology of early-stage CRC in relation to *VDR* and other pertinent SNPs remains unclear. The development of CRC is significantly influenced by gene–gene, gene–diet, and gene–environment interactions, as well as lifestyle factors. The present study provides evidence that the relative expression levels of *VDR*, *RXR*, *CYP27B1*, and *CYP24A1* were significantly elevated following high-dose VD_3_ supplementation for three months. Interestingly, none of the SNPs or haplotypes within the *VDR* gene, *CYP27B1* (rs10877012, rs6013897, rs158552), or *CYP24A1* (rs17217119) were associated with colorectal cancer susceptibility.

Several studies have identified multiple SNPs associated with *VDR* function, with five common variants—FokI, BsmI, ApaI, TaqI, and Tru9I—most frequently reported in association studies. Among the five SNPs, only FokI is a nonsynonymous SNP located at the 5′ end of exon 2. Only variation in the FokI SNP caused a change in the translation start site, which in turn resulted in a smaller protein with increased activity. The other four SNPs are nonsynonymous variations. BsmI, Tru9I, and ApaI are inside intron 8, and only TaqI is in exon 9 [28].

**Figure 2:**
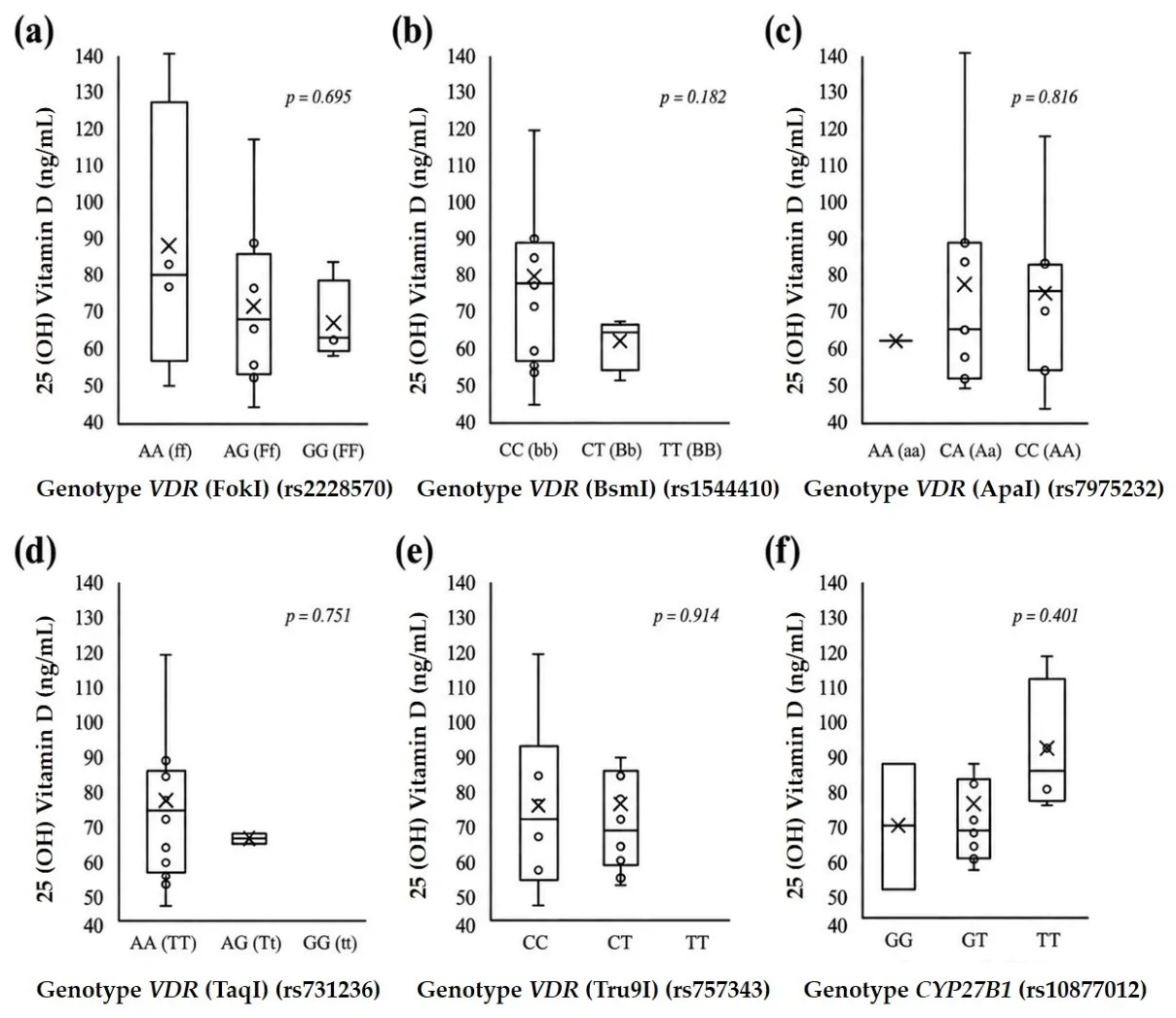
Comparison of 25(OH)D_3_ level according to genotypes of the following SNPs: (**a**) rs2228570 (FokI), (**b**) rs1544410 (BsmI), (**c**) rs7975232 (ApaI), (**d**) rs731236 (TaqI), and (**e**) rs757343 (Tru9I) in the *VDR* gene and (**f**) rs10877012 in *CYP27B1* gene in colorectal cancer patients after 3 months of VD_3_ supplementation (8000 IU/day). Data are presented as median and ranges of serum 25(OH)D_3_ level (ng/mL). The comparison between genotypes was designed based on the nonparametric Kruskal–Wallis test and Bonferroni correction. X represented for median.

FokI polymorphism (rs2228570) is a C/T transition that addresses the start codon of the *VDR* gene, also known as a functional polymorphism because it changes the structure and function of the encoded protein. This allelic change resulted in two structural receptor proteins, which showed different abilities to induce transcription of the vitamin D-dependent gene [29]. The FokI F allele was reported to change the location of the start codon later than the f allele, generating a smaller protein with higher transcription activity [30]. The FokI C nucleotide is referred to as allele F, while the T nucleotide is defined as allele f. In this study, the FokI SNP polymorphism was not significantly associated with CALF levels or gene expression. However, a trend was observed in which the ff genotype exhibited lower CALF levels compared with the Ff and FF genotypes (Figure 2a). When examining correlations with gene expression levels, the ff genotype demonstrated a greater fold change (1.32) compared with the Ff and FF genotypes and exhibited the highest fold change among the SNPs analyzed in this study (Table 4). These findings suggest that the responsiveness to vitamin D supplementation, reflected in both the serum vitamin D levels and *VDR* gene expression, appears to follow the order ff > Ff > FF. This pattern may indicate a potential benefit of vitamin D supplementation in modulating *VDR* gene expression through FokI polymorphism.

BsmI SNP (rs1544410) consists of an A-to-G nucleotide substitution. However, this polymorphism does not change the encoded protein, but it does affect the level of *VDR* transcription, mRNA stability, and posttranscriptional modification [29] by influencing transcription stability [18]. The BsmI A nucleotide corresponds to allele B, and the G nucleotide corresponds to allele b. In this study, the BsmI variant was not significantly associated with serum vitamin D levels or *VDR* gene expression. However, a trend was observed in which the BsmI SNP appeared to correlate with serum vitamin D levels following three months of supplementation. Individuals with the bb genotype exhibited the highest CALF levels, followed by those with the Bb genotype. Notably, the BB genotype was not detected among any participants (Figure 2b). This finding is consistent with Ghiasvand et al. [17], who reported that the BsmI bb genotype is more prone to increase CALF in response to vitamin D supplements compared to the BB+Bb genotype, and they suggested that homozygous bb genotype carriers are favored for having greater receptor availability and benefit from the evaluating effects on CALF levels. Furthermore, Xia et al. [31] reported that the bb genotype has a higher rate of *VDR* expression compared with subjects with the BB and Bb genotypes. In this study, BsmI SNP and *VDR* gene expression results were analyzed and showed a trend corresponding to Xia et al. [31]. As shown in Table 4, the bb genotype has a higher fold change at 1.05 than the Bb and BB genotypes. These findings suggest that the homozygous bb genotype may confer greater benefit from vitamin D supplementation through enhanced effects on serum vitamin D levels. In contrast, the presence of the B allele appears to attenuate *VDR* gene expression, thereby reducing the availability of vitamin D to exert its modulatory functions.

The ApaI polymorphism (rs7975232) consists of a C to A substitution polymorphism. The C nucleotide is referred to as allele “A,” while the A nucleotide is referred to as allele “a.” In these experiments, no association was found between the ApaI polymorphism and the CALF level or the expression of the *VDR* gene. However, this association was reported in other studies. Ghiasvand et al. [17] reported that the ApaI polymorphism aa genotype is more prone to increase CALF levels in response to vitamin D supplementation than genotypes AA and Aa. In addition, vitamin D supplementation in the dose–response analysis indicates that a dose of less than 30,000 IU/week can significantly decrease the serum CALF level, while a dose of more than 30,000 IU/week to 50,000 IU/week can significantly increase the serum level. In contrast to Al-Ghafari et al. (2020) [32], the present results revealed that the ApaI SNP aa genotype showed the lowest CALF level, and the Aa genotype showed the highest level after 3 months of VD_3_ supplementation (Figure 2c). The ApaI SNP has no impact on the amino acid sequence or structure of the VDR protein; however, it could alter the stability of the *VDR* mRNA and/or interfere with *VDR* transcription. Untranslated regions can modulate levels of gene expression, most notably through regulation of mRNA stability. Reduced mRNA stability, and, consequently, diminished translation of the VDR protein, leads to attenuated cellular responses to vitamin D. In this context, the ApaI polymorphism may act as an intronic enhancer, potentially mediating alternative splicing of *VDR* mRNA and/or functioning as a regulatory element that augments gene transcription [33].

The TaqI polymorphism (rs731236) is a T-to-C substitution. This polymorphism occurs in a CpG island, resulting in an influence on the methylation process. The T nucleotide is defined as allele T, while the C nucleotide corresponds to allele “t.” In this study, no association was observed between the TaqI polymorphism and CALF level and/or expression levels of the *VDR* gene in both the intervention and control groups. Although the TaqI SNP showed no significant association overall, analysis of its correlation with CALF25(OH)D_3_ levels after three months of VD_3_ supplementation revealed a trend in which the TT genotype exhibited a greater increase in serum vitamin D compared with the Tt genotype (Figure 2d). Notably, the tt genotype was not represented in this study population. These findings are consistent with those of Ghiasvand et al. [17], who reported that individuals with the TaqI tt genotype exhibited significantly lower vitamin D levels in response to supplementation, whereas the TT genotype displayed higher levels compared with both Tt and tt genotypes.

The TT of the Tru9I of the *VDR* gene (rs757343) was unable to be determined in these participants. Polymorphism analysis revealed no significant correlation between the expression of the *VDR* gene and serum vitamin D level. In this study, after 3 months of vitamin D supplementation in the intervention group, participants with the CC and CT genotypes showed similar serum vitamin D levels, averaging 73.58 and 74.44 ng/mL, respectively, as shown in Table 5 and Figure 2e. These results suggest that allelic variation in the Tru9I SNP does not substantially enhance *VDR* gene expression when comparing the wild-type and mutant alleles. This observation aligns with the findings of Shawi et al. [34], who reported that the Tru9I SNP does not alter the amino acid sequence of the encoded protein, but may influence gene expression by modulating mRNA stability or through linkage disequilibrium.

The *CYP27B1* gene, which encodes the 1α-hydroxylase enzyme and converts CALF to its active form CAL. The SNP rs10877012 polymorphism of *CYP27B1* is located on the 5′ untranslated region and was reported to be associated with a reduced vitamin D level [35]. Consistent with findings on SNPs in *VDR*-related genes, the present study demonstrated that the rs10877012 polymorphism had no significant impact on serum vitamin D levels or *CYP27B1* gene expression in CRC patients receiving VD_3_ supplementation compared with the control group. The allelic distribution of *CYP27B1* is presented in Table 3. Analysis of serum vitamin D levels after three months of high-dose vitamin D supplementation in the intervention group revealed no association with the rs10877012 allelic variants. Interestingly, these results revealed a trend of increasing serum vitamin D levels across different genotypes of the polymorphism. Patients carrying the TT genotype exhibited the highest serum vitamin D levels, with a median of 85.48 ng/mL after three months of supplementation, followed by the GT and GG genotypes, respectively (Figure 2f). Furthermore, analysis of gene expression showed that the TT genotype demonstrated a greater fold change (0.94) compared with the GT and GG genotypes (Table 4).

## 5. Conclusions

The findings obtained in these studies indicate that the upregulation of *VDR* and related genes in early-stage CRC patients was independent of *VDR* and other relevant SNPs. High-dose vitamin D_3_ supplementation for 3 months was associated with higher expression of *VDR*, *RXR*, and *CYP27B1* compared with the control group under the conditions of this study. Nevertheless, these gene expression changes were not associated with corresponding genetic polymorphisms. Therefore, the present findings do not provide evidence that polymorphisms in *VDR*, *CYP27B1*, and *CYP24A1* influence the effects of vitamin D intake in patients with early-stage CRC. However, these findings should be interpreted cautiously because of the limited sample size and statistical power, and they require validation in larger, independent studies.

### Limitation of the Study

One limitation of this study is the relatively small sample size, particularly for the gene expression analyses (*n* = 24–25) and the vitamin D supplementation analysis (*n* = 16). In addition, several SNPs had low minor genotype frequencies, resulting in small numbers of participants in some genotype groups. Although rare genotypes were combined under a dominant genetic model where appropriate to improve statistical stability, the statistical power to detect small-to-moderate genetic effects remained limited. Consequently, the observed median expression ratios (fold changes) should be interpreted as exploratory effect estimates rather than precise measures of the magnitude of the observed effect. Therefore, the absence of statistically significant associations should not be interpreted as evidence of the absence of a biological effect, and these findings require validation in larger, independent cohorts.

## Figures and Tables

**Table 1:** Primers used for gene expression levels.

Gene	Primer Sequence (5′ to 3′)	Size (bp)	Accession No./ Reference
*VDR*	F: GACTTTGACCGGAACGTGCCC	227	NM000376
	R: CATCATGCCGATGTCCACACA		
*RXR*	F: GGACATGCAGATGGACAAGAC	71	Höbaus et al., 2013 [26]
	R: CCTTGGAGTCAGGGTTAAAGAG		
*CYP27B1*	F: TGGCCCAGATCCTAACACATTT	73	Horváth et al., 2010 [27]
	R: GTCCGGGTCTTGGGTCTAACT		
*CYP24A1*	F: GGAGATCAAACCGTGGAAGG	145	NM_001424343.1
	R: GTTGTCCAGCTTCATCACTTCC		
*β*-Actin	F: TGGCTCCCGAGGAGCAC	97	Horváth et al., 2010 [27]
	R: TTGAAGGTCTCAAACATGATCTGG		

**Table 2:** Primer names, primer sequences, target genes, and product sizes used in DNA sequencing reactions for SNP genotyping.

Primer Name	Target Gene	SNP	Sequences (5′-3′)	Product Size (bp)
SQ1-Forward SQ1-Reverse	*VDR*	rs7975232 rs731236	ACAAGGGGCGTTAGCTTCAT GAGGGATGGACAGAGCATGG	501
SQ2-Forward SQ2-Reverse	*VDR*	rs1544410 rs757343	AATGAGAGAAGGCTGGCTGG GGACAAAGACCTGCTGAGGG	605
SQ3-Forward SQ3-Reverse	*VDR*	rs2228570	GGGCTCCCTTCATGGAAACA CTGAGCCAGCTATGTAGGGC	434
SQ4-Forward SQ4-Reverse	*CYP24A1*	rs6013897 rs158552 rs17217119	CCTTTTATTTACCACTTGTCTGAG AGATGTGCAGATCACAGATTGTT	487
SQ5-Forward SQ5-Reverse	*CYP27B1*	rs10877012	AGCTGACTCGGTCTCCTCTG ACCATCCTCCTGTCCTCTCC	714

**Table 3:** The frequency of *VDR*, *CYP27B1*, and *CYP24A1* SNPs, allele/genotypes at the polymorphic site, and test of HWE between the intervention (*n* = 16) and control groups (*n* = 11).

	*VDR* TaqI (rs731236)				*VDR* ApaI (rs7975232)			
	SNP location	Nucleotide change	SNP alleles	*p*, HWE	SNP location	Nucleotide change	SNP alleles	*p*, HWE
	Exon 9	C>T	T>t		Intron 8	A>C	A>a	
n (%)	CC (TT)	CT (Tt)	TT (tt)		AA (AA)	AC (Aa)	CC (aa)	
Intervention	0 (0.00)	2 (12.5)	14 (87.5)	1.000	2 (12.5)	7 (43.75)	7 (43.75)	1.000
Control	1 (9.09)	0 (0.00)	10 (90.9)	0.348	1 (9.09)	5 (45.45)	5 (45.45)	1.000
	*VDR* BsmI (rs1544410)				*VDR* FokI (rs2228570)			
	SNP location	Nucleotide change	SNP alleles	*p*, HWE	SNP location	Nucleotide change	SNP alleles	*p*, HWE
	Intron 8	A>G	B>b		Exon 2	C>T	F>f	
n (%)	AA (BB)	AG (Bb)	GG (bb)		CC (FF)	CT (Ff)	TT (ff)	
Intervention	0 (0.0)	4 (25.0)	12 (75.0)	1.000	4 (25.0)	8 (50.0)	4 (25.0)	1.000
Control	1 (9.09)	0 (0.0)	10 (90.91)	0.348	3 (27.27)	6 (54.54)	2 (18.19)	1.000
	*VDR* Tru9I (rs757343)				*CYP27B1* (rs10877012)			
	SNP location	Nucleotide change	SNP alleles	*p*, HWE	SNP location	Nucleotide change	SNP alleles	*p*, HWE
	Intron 8	C > T	-		Promoter	G>T	T>t	
n (%)	CC	CT	TT		GG (TT)	GT (Tt)	TT (tt)	
Intervention	6 (37.5)	10 (62.5)	0 (0.0)	0.248	2 (12.50)	10 (62.50)	4 (25.00)	0.597
Control	6 (54.54)	5 (45.45)	0 (0.0)	0.535	2 (18.18)	5 (45.45)	4 (36.36)	1.000
	*CYP24A1* (rs6013897) P450 mitochondrial cytochrome				*CYP24A1* (rs158552) P450 mitochondrial cytochrome			
	SNP location	Nucleotide change	SNP alleles	*p*, HWE	SNP location	Nucleotide change	SNP alleles	*p*, HWE
		T>A	-			C>T	-	
n (%)	TT	TA	AA		CC	CT	TT	
Intervention	15 (93.75)	1 (6.25)	0 (0.0)	1.000	0 (0.0)	2 (12.5)	14 (87.5)	1.000
Control	11 (100.00)	0 (0.0)	0 (0.0)	1.000	0 (0.0)	1 (9.09)	10 (90.9)	1.000
	*CYP24A1* (rs17217119) P450 mitochondrial cytochrome							
	SNP location	Nucleotide change	SNP alleles	*p*, HWE				
		A>G	-					
n (%)	AA	AG	GG					
Intervention	16 (100.00)	0 (0.0)	0 (0.0)	1.000				
Control	11 (100.00)	0 (0.0)	0 (0.0)	1.000				

SNP, single-nucleotide polymorphism; HWE, Hardy–Weinberg equilibrium; *p* > 0.05 indicates no significant difference in the distribution of genotype according to HWE.

**Table 4:** Association between *VDR*, *CYP24A1*, and *CYP27B1* expressions and the SNPs of these genes in the early stages of CRC in patients.

Gene	SNPs	Genotype	Frequency n (%)	Median (2^^ΔCt^)	Fold Change *	*p*-Value **
*VDR*	rs2228570 FokI	AA	4 (16.67)	0.222594	1.32	1.000
		AG + GG	20 (83.33)	0.168116		
	rs1544410 BsmI	CC	20 (83.33)	0.181268	1.05	0.877
		CT + TT	4 (16.67)	0.173259		
	rs7975232 ApaI	CC	10 (41.67)	0.217450	1.26	0.482
		CA + AA	14 (58.33)	0.173259		
	rs731236 TaqI	AA	22 (91.67)	0.171502	0.72	0.602
		AG + GG	2 (8.33)	0.236776		
	rs757343 Tru9I	CC	9 (37.50)	0.166359	0.85	0.929
		CT + TT	15 (62.50)	1.196178		
*CYP27B1*	rs10877012	GG	2 (8.33)	1.060000	0.94	0.277
		GT + TT	22 (91.67)	1.130000		
*CYP24A1*	rs6013897	TT	23 (95.83)	2.180000	0.99	0.942
		TA + AA	1 (4.17)	2.180953		
	rs158552	CC	0 (0.00)	ND	ND	ND
		CT + TT	24 (100.00)	ND		
	rs17217119	AA	24 (100.00)	ND	ND	ND
		AG + GG	0 (0.00)	ND		

Abbreviations: ND, not determined; SNPs, single-nucleotide polymorphisms. * The fold change was calculated by dividing the wild-type value by the mutant value. ** Nonparametric analysis of variance (the Wilcoxon signed-rank test).

**Table 5:** Associations between *VDR*, *CYP27B1*, and *CYP24A1* SNPs and serum vitamin D level at 3 months after VD_3_ supplementation in intervention group (*n* = 16).

Gene	SNP	Genotype	Frequency *n* (%)	Serum Vitamin D level VD_3_ Supplementation in Intervention Group (Mean ± SD)	*p*-Value
*VDR*	rs2228570 FokI	AA	4 (25.00)	87.65 ± 38.36	0.695
		AG	8 (50.00)	71.18 ± 23.72	
		GG	4 (25.00)	66.48 ± 11.37	
	rs1544410 BsmI	CC	12 (75.00)	78.83 ± 27.91	0.182
		CT	4 (25.00)	59.98 ± 6.79	
		TT	0 (0.00)	ND	
	rs7975232 ApaI	AA	2 (12.50)	62.35 ± 0.63	0.816
		CA	7 (43.75)	76.85 ± 31.78	
		CC	7 (43.75)	74.74 ± 23.59	
	rs731236 TaqI	AA	14 (87.50)	75.56 ± 27.09	0.751
		AG	2 (12.50)	64.00 ± 1.69	
		GG	0 (0.00)	ND	
	rs757343 Tru9I	CC	6 (37.50)	73.58 ± 26.06	0.914
		CT	10 (62.50)	74.44 ± 26.61	
		TT	0 (0.00)	ND	
*CYP27B1*	rs10877012	GG	2 (12.50)	79.75 ± 4.17	0.401
		GT	10 (62.50)	68.45 ± 21.74	
		TT	4 (25.00)	85.475 ± 39.17	
*CYP24A1*	rs6013897	TT	15 (93.75)	74.71 ± 26.31	0.914
		TA	1 (6.25)	65.20 ± 0.00	
		AA	0 (0.00)	ND	
	rs158552	TT	14 (87.50)	75.79 ± 26.81	0.525
		CT	2 (12.50)	62.45 ± 10.81	
		CC	0 (0.00)	ND	
	rs17217119	AA	16 (100.00)	74.12 ± 26.53	1.00
		AG	0 (0.00)	ND	
		GG	0 (0.00)	ND	

## Data Availability

The original contributions presented in this study are included in the article. Further inquiries can be directed to the corresponding author.

## Abbreviations

The following abbreviations are used in this manuscript:

*VDR*	Vitamin D receptor
*RXR*	Retinoid X receptor
*CYP27B1*	25-hydroxyvitamin D-1α-hydroxylase
*CYP24A1*	25-hydroxyvitamin D-24-hydroxylase
SNP	Single-nucleotide polymorphism
CRC	Colorectal cancer
cDNA	Complementary DNA
HWE	Hardy–Weinberg equilibrium
